# Cost-Effectiveness of Introducing the SILCS Diaphragm in South Africa

**DOI:** 10.1371/journal.pone.0134510

**Published:** 2015-08-21

**Authors:** Aurélia Lépine, Neeti Nundy, Maggie Kilbourne-Brook, Mariana Siapka, Fern Terris-Prestholt

**Affiliations:** 1 Global Health & Development department, London school of hygiene & Tropical Medicine, London, United Kingdom; 2 PATH, Seattle, Washington, United States of America; State University of Maringá/Universidade Estadual de Maringá, BRAZIL

## Abstract

**Background:**

Though South Africa has high contraceptive use, unintended pregnancies are still widespread. The SILCS diaphragm could reduce the number of women with unmet need by introducing a discreet, woman-initiated, non-hormonal barrier method to the contraceptive method mix.

**Methods:**

A decision model was built to estimate the impact and cost-effectiveness of the introduction of the SILCS diaphragm in Gauteng among women with unmet need for contraception in terms of unintended and mistimed pregnancies averted, assuming that the available contraceptives on the market were not a satisfying option for those women. Full costs were estimated both from a provider’s and user’s perspective, which also accounts for women’s travel and opportunity cost of time, assuming a 5% uptake among women with unmet contraceptive need. The incremental cost-effectiveness ratio is computed at five and 10 years after introduction to allow for a distribution of fixed costs over time. A probabilistic sensitivity analysis was conducted to incorporate decision uncertainty.

**Results:**

The introduction of the SILCS diaphragm in Gauteng could prevent an estimated 8,365 unintended pregnancies and 2,117 abortions over five years, at an annual estimated cost of US$55 per woman. This comes to a cost per pregnancy averted of US$153 and US$171 from a user’s and provider’s perspectives, respectively, with slightly lower unit costs at 10 years. Major cost drivers will be the price of the SILCS diaphragm and the contraceptive gel, given their large contribution to total costs (around 60%).

**Conclusions:**

The introduction of the SILCS diaphragm in the public sector is likely to provide protection for some women for whom current contraceptive technologies are not an option. However to realize its potential, targeting will be needed to reach women with unmet need and those with likely high adherence. Further analyses are needed among potential users to optimize the introduction strategy.

## Introduction

There is an urgent need to narrow the contraceptive gap in South Africa. One way to achieve this would be the introduction of new contraceptive products. Though contraceptive prevalence in South Africa among sexually active women aged 15–49 is among the highest in Africa, with around 62% of women using modern contraception according to the 2003 Demographic and Health Survey (DHS) [1], women still report high levels of unwanted pregnancy [1]. According to the most recent DHS data, only 48% of women wanted their last child [1]. These unintended pregnancies place a significant burden on health systems and families, generating a high cost for society. Children from unwanted conception are more likely to suffer from malnutrition, mothers are at greater risk of depression and physical abuse, and parents may invest less in the education of unwanted children [1]. Unintended pregnancies are also costly when they result in abortions. Even though abortion is legal in South Africa, many women, especially young women, undergo unsafe abortion because they do not access services at registered health facilities [2, 3].

Modern contraception methods include combined oral contraceptives, progestogen-only pill, implants, progestogen-only injectables, monthly injectables, intrauterine device, male and female condom, male and female sterilization, lactational amenorrhea, diaphragm and emergency contraception. (http://www.who.int/mediacentre/factsheets/fs351/en/) From the DHS data, the main reasons for not using modern contraception among South African women aged 15–49 who are in an union and who are sexually active were health concerns (25%), cost too much (14%), fear of side effects (5%) and the interference with body processes (8%) [1], addressing those factors could increase the uptake of existing contraceptive methods. In a systematic review investigating the barriers to modern contraceptives in low and middle income countries, it was highlighted that although condom use could appear more attractive than hormonal methods, its use was limited by its association with HIV/AIDS together with the fact that its use was under the partner control [4]. This suggests that a user-initiated, non-hormonal barrier method, with few side effects, would address some of the key reasons why women with an unmet need do not use existing methods [5] and this also justifies why the comparator for our cost-effectiveness analysis of the SILCS diaphragm is a situation where women still have unmet need for contraception.

This cost-effectiveness analysis is designed to inform stakeholders who are interested in including the SILCS diaphragm, a new contraceptive diaphragm, to expand the method mix to address unmet need for contraception. In clinical studies, the SILCS diaphragm has been shown to have similar effectiveness as a traditional diaphragm when both are used with contraceptive gel [6, 7]. According to the WHO Family Planning handbook classification on contraceptive effectiveness the diaphragm is considered moderately effective [8]. Data from South Africa indicate that the traditional diaphragm is not an attractive modern contraceptive method for South African women as none of the women in the sample is using this mode of contraception [1]. The SILCS diaphragm was developed through a user-centered process which focused on experience from women and men in South Africa and other countries to validate the design. The SILCS diaphragm provides certain advantages over traditional diaphragms. The single-size design should be easy to supply, and the SILCS has several special features that make it easy to insert and remove. While SILCS will likely be offered by a trained nurse during early introduction, unlike other diaphragms it does not require a pelvic exam to determine diaphragm size. Similar to other diaphragms, it is non-hormonal, has few (if any) side effects and is reusable for at least two years. The SILCS diaphragm provides more discreet protection than male or female condoms so may require less partner negotiation. However, little information is available about diaphragm cost-effectiveness in low-resource settings as diaphragms have not been included in the family planning programs in developing countries in recent decades. Therefore, we developed a model to better understand the cost-effectiveness of the SILCS diaphragm using cost data from the Gauteng province in South Africa. The choice of the region was influenced by data availability and a potential first region for introduction.

## Methods

Our model simulates the incremental cost-effectiveness ratio (ICER) of introducing the SILCS diaphragm among sexually active women with unmet need for contraception in Gauteng Province, South Africa. This model estimated impact in terms of averted unintended and mistimed pregnancies, i.e. pregnancies that are unintended in the present but that are desired in the future, following Trussell et al. [9, 10]. Provider and provider plus user costs were estimated. Provider plus user costs include in addition to the costs incurred at the provider, transportation costs and opportunity cost of time of the user. Averted costs were included in the analysis by estimating the adverse outcomes averted. Costs and effects are discounted using a 3% discount rate. Finally, we conducted a probabilistic sensitivity analysis to explore the effect of uncertainty in our model. Here follows a brief description of the model and inputs, with full input and assumptions provided in S1 Table. Inputs were obtained both from published literature, computed from the 2003 DHS data set [1], or obtained from discussions with experts in the absence of published data. For the published literature, our search strategy, conducted in July 2013, identified 33 potentially relevant articles that were in English and published in or after 2006 in South Africa. Out of these studies, nine studies had costing information relevant to our search (see S1 Text for further information on the search strategy).

### The setting and introduction scenario

Gauteng Province is largely urban (97%) and consists of the major cities of Johannesburg and Pretoria, with feeder cities and townships. As of 2003, there was a large public health clinic network with 295 primary health care facilities [11], within which contraception is provided free of charge in public facilities [12]. We modeled that the SILCS diaphragm is introduced by the public sector, supported by a mass media campaign [13], and includes the three-year rollout of provider training in all public health facilities as well as subsequent refresher trainings every three years [14]. We assume that the SILCS diaphragms are provided to women by a nurse within a 30-minute visit, including counseling and training on correct use. Women are asked to return to a health facility every two years for a new device.

For this analysis, eligible SILCS diaphragm users are defined as married or women in a union aged 15 to 49 who want to stop or delay childbearing but are not using modern contraception (e.g. women with unmet need). Projections of population growth and unmet need for contraception were estimated using United Nations models [15, 16]; further details are provided in S2 Text. We assume a 5% uptake of the diaphragm among women with unmet need from 2013 to 2023. We present the results for the cost-effectiveness analysis at five and 10 years, which is in line with the current policy decision timelines.

### Impact: model of averted unintended pregnancies

The impact is estimated by the number of pregnancies averted by the diaphragm, i.e. the difference between the likelihood of getting pregnant per year without any modern contraception and the likelihood of getting pregnant per year when using the diaphragm, multiplied by the number of women using the diaphragm. The likelihood of getting pregnant without using contraception was estimated as 40% based on DHS data for South Africa [1]. The annual probability of pregnancy while using the SILCS diaphragm with a contraceptive gel is 17.8% for typical use and 13.7% for perfect use per year [7]. The model assumes either perfect use or typical use, and both results are presented.

### Costs

We calculated the incremental costs of public-sector SILCS diaphragm distribution from a provider perspective (i.e. the health systems costs). Incremental costs are calculated by subtracting averted costs from total costs, averted costs being defined as the costs associated with unwanted and mistimed births avoided by programming SILCS diaphragms.

Provider costs of the SILCS diaphragm distribution include both fixed and variable costs. Fixed provincial-level costs refer to a mass media campaign. Facility fixed costs include the initial training and refresher training that occurs every three years. Variable costs (defined as costs that vary with the number of users) include product cost and counseling cost. In addition, variable costs include overhead costs that include a facility markup to capture direct health facilities costs beyond the estimation of required staff time [17] and health system costs (assumed to represent half of the health facility markup) to capture the costs of upper management, logistics, etc., following Eaton et al. [18]. The cost of the SILCS diaphragm delivered to South Africa for public-sector distribution is estimated at US$5.19 per diaphragm (Kessel, personal communication, 2013) including regulatory fees, shipping, taxes, and customs. The diaphragm is estimated to have a two-year usable life. Counseling is estimated as a 30-minute session with a nurse every two years, when a new diaphragm is provided. Gel use is estimated at 4 ml per dose, two sex acts per week [19], and 10% wastage, requiring about 6.5 tubes of 70 ml per year. At US$3.69 per tube (Kessel, personal communication) this results in a yearly gel cost of US$25.83. Since we assume SILCS will be distributed by the public sector, the costs of the diaphragm and gel will be consider a provider cost.

User costs of diaphragm collection includes transport costs to a public health facility in Gauteng for diaphragm collection visits once every two years (gel collection is assumed to be integrated into routine activities, thus does not contribute extra user costs) and the opportunity cost of labor (e.g. loss of income, assuming here a loss of three days of labor on average due to delivery) every two years [20]. These costs vary by location type [20] and are incorporated using Gauteng’s urban/rural population distribution [21] for estimates of transport and opportunity costs. Total mass media and training cost represents only 6% and 4% of total costs at five and 10 years, respectively, while the diaphragm and gel costs account for about 60% of total costs (S1 Fig).

Table 1 presents the estimates of the provider and provider plus user costs per 1,000 users. The variable cost per woman is about US$109 for the 2-year SILCS diaphragm lifespan or US$55 per woman per year.

**Table 1:** Diaphragm unit cost per 1,000 users (2011US$).

	In US$
*Provider costs*	
*Capital costs*	
Mass media costs	196
*Fixed costs per health facility*	
Training pre-intervention	547
Refresher training costs every three years	164
*Variable costs*	
Counseling costs per diaphragm lifespan	7,770
Facility and system markup per diaphragm lifespan	15,530
Diaphragm and contraceptive gel costs per diaphragm lifespan	67,700
*User costs*	
Transport costs over two years (weighted average using rural and urban composition)	14,770
Opportunity costs over two years (weighted average using rural and urban composition)	3,350
*Provider variable costs and user costs*	
Total cost per diaphragm lifespan	109,120

Preventing unintended pregnancies generates averted costs that are estimated by the cost of averted antenatal care (ANC) and delivery. We assumed that 92% of pregnant women who continue their pregnancy will access ANC and a facility-based delivery [21]. ANC visits are modeled as 30 minutes of nurses’ time. Women in South Africa complete, on average, 3.8 visits [22], thus we assume four prenatal visits per woman. Delivery cost is the weighted average of the cost of different delivery outcomes (delivery of a healthy child, low-birth weight, neonatal death, stillbirth, and miscarriage) by population outcomes [23]. This model does not estimate averted costs that occur beyond the costs associated with birth or abortion such as the cost of raising a child or the eventual loss of future income due to school dropout of teen mothers.

All costs are presented in 2011 US Dollars (US$). Non-tradable goods (e.g., labor costs, training cost, transport cost, and opportunity cost of time) were inflated using the local currency inflation rates then converted into US$ [24]. For tradable goods (e.g., product cost), the cost was inflated using the US$ inflation rate [24].

**Fig 1:**
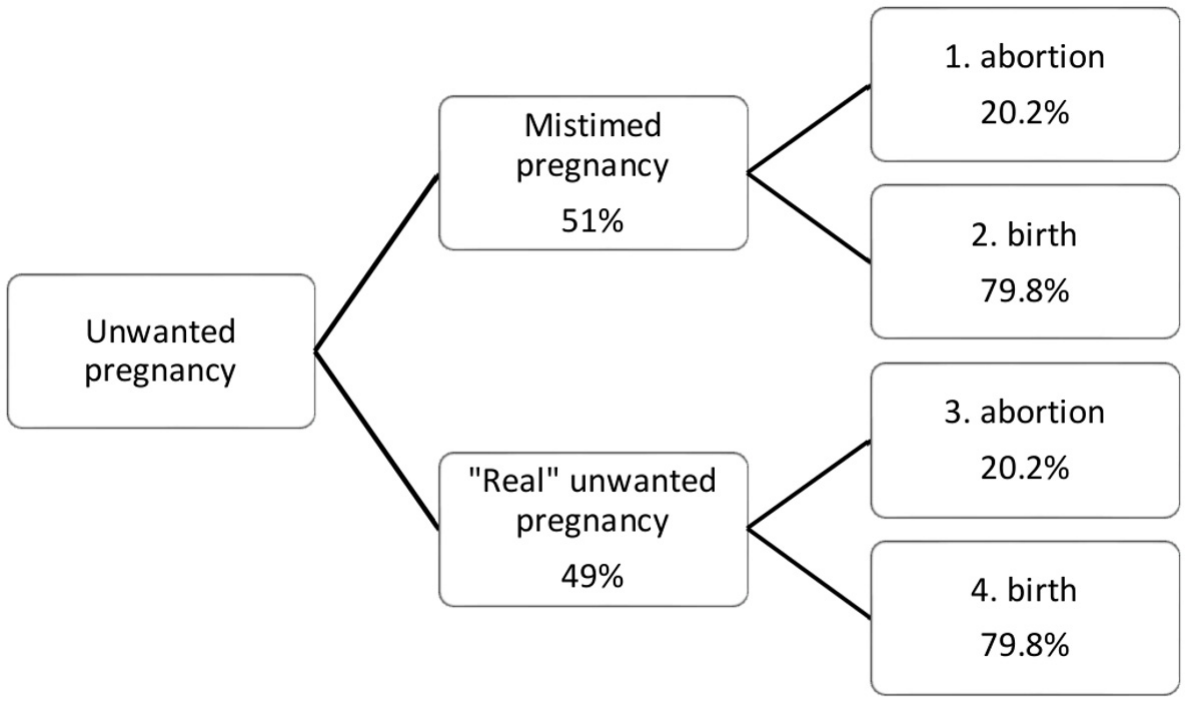
Fig 1 presents the decomposition of unwanted pregnancies into mistimed and “real” unwanted pregnancies as well as the associated abortion rate for each category.

We distinguish between two types of unwanted births: “real” unintended births that results in averted costs and mistimed unintended births that are the births that occur too early but ideally would have occurred in the future [9]. In order to estimate the proportion of mistimed births, we looked at the proportion of women whose pregnancy was unintended and who declare that they would have liked to have this birth later. Among the women who did not want their last child (n = 1,434 representing 52% of total women), 49% declare that the last child was unwanted and 51% declare that they would have liked to have this birth in the future [21]. Following Trussell [9], we assume that a mistimed birth would have occurred 2 years later. Additionally, we incorporate the 10.5% abortion rate [25], assuming that abortion among women wanting pregnancy is nil and that the abortion rate is equal among mistimed and unwanted births; the abortion rate among the women with undesired pregnancy is estimated at 20.2% (10.5*1/0.52). Abortion cost was estimated to be US$76 at the facility level in the literature [26]. The proportion of averted pregnancies that would result in “real” averted pregnancies, delayed pregnancies, and abortions is presented in Fig 1, and related costs are used to compute the total averted costs in Table 2, calculations are detailed in endnote. Including mistimed pregnancies and abortions resulted in lower averted costs. By including mistimed pregnancies, the provider cost of an averted pregnancy is US$126 (instead of US$368 and US$265, respectively), adding user costs to provider cost leads to a unit cost of an averted pregnancy of US$189.

**Table 2:** Averted costs of averted pregnancy including mistimed pregnancies and abortions.

Type of averted costs	Facility and patient costs (2011US$)	Facility costs (2011US$)
1. Total cost of abortion for mistimed birth	18	7.8
2. Total cost of delivery and antenatal care including mistimed birth (cf Trussell, 2008)	8.6	6.2
3. Total cost of abortion for real unintended pregnancies	17.5	7.6
4. Total cost of birth for real unintended pregnancies	144.7	104.3
Total averted cost per pregnancy averted	188.7	125.9

1. Abortion cost*Proportion of mistimed pregnancy*Proportion of abortion

2. Delivery cost*(1-(1/(1+discount rate)^Nb of years birth is delayed^))*Proportion of mistimed pregnancy*Proportion of birth.

3. Abortion cost*Proportion of “real” unwanted pregnancy*Proportion of abortion.

4. Delivery cost*Proportion of “real” unwanted pregnancy*Proportion of birth

Finally, we conducted a probabilistic sensitivity analysis (PSA) in order to investigate the effect of uncertainty on our results. Parameters assumed to contain uncertainty as well as their associated mean, standard error, and distribution function are presented in S2 Table.

## Results

**Fig 2:**
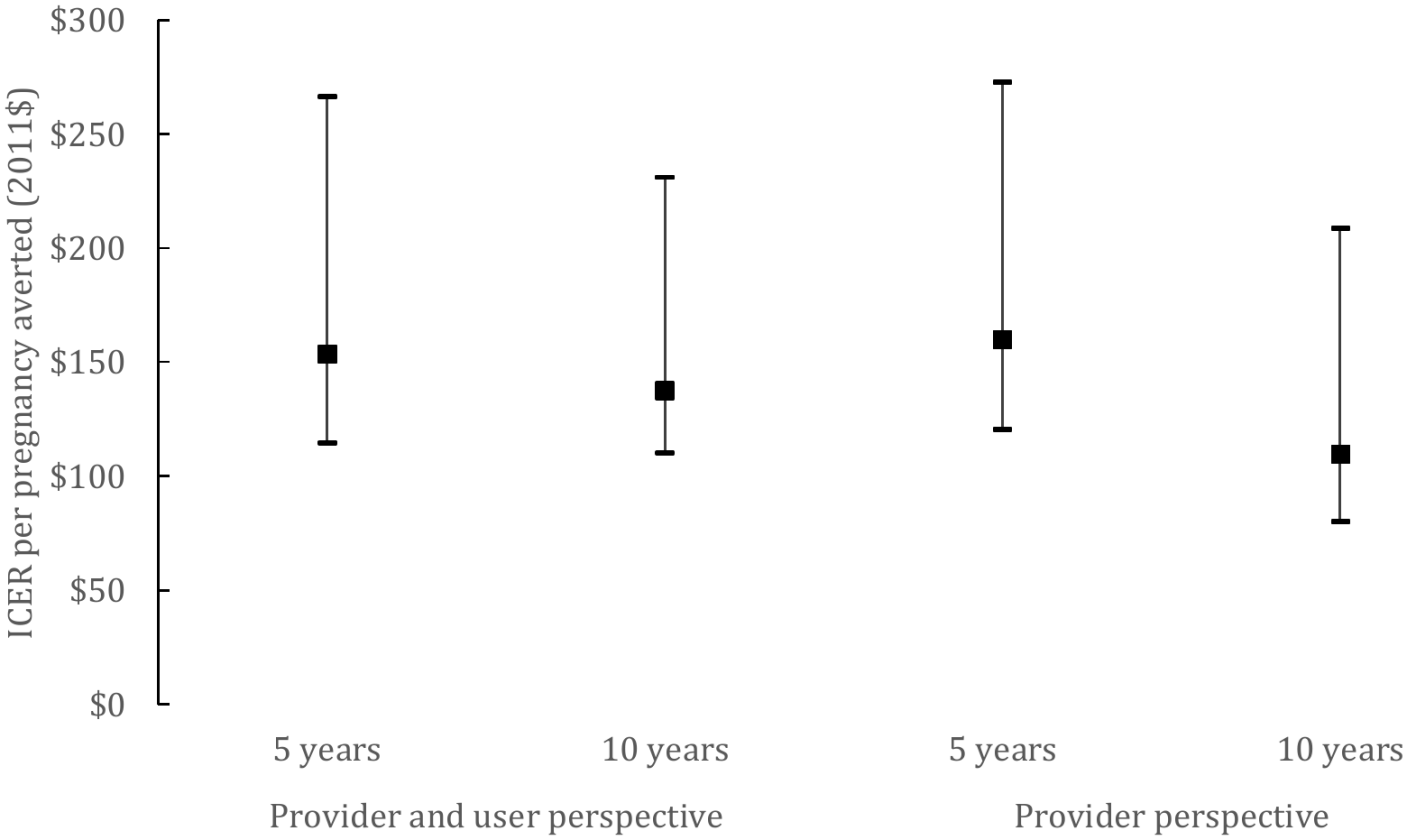
Fig 2 presents the ICER per pregnancy averted in 2011 USD assuming a typical use.

We estimated the ICER of SILCS introduction, compared to the status quo i.e. assuming that SILCS users are not on a contraceptive, in terms of cost per pregnancy averted from the provider and user perspectives assuming typical and perfect diaphragm use and three population growth assumptions (low, medium, and constant) at five and 10 years post introduction (see S3 Table for more details on the model inputs and outputs). Fig 2 provides the facility- and user-based ICER for typical use over different time horizons, and the confidence intervals represent different scenarios to model population projection and the unmet need for contraception in the future. Considering median assumptions and a provider’s perspective, Fig 2 shows the estimated cost of introducing the SILCS diaphragm of US$171 per pregnancy averted (US$132-US$284) at five years and US$119 per pregnancy averted (US$89-US$218) at 10 years. Inclusion of user costs (transport cost, opportunity cost of time, and the payment for substitute labor) was added to the provider costs to estimate costs from a user approach. The costs considering the user perspective would be US$153 per pregnancy averted at five years and US$138 at 10 years. The ICER is strongly dependent on assumptions regarding population growth and the assumption regarding the proportion of women with unmet need for contraception. This explains the difference between lower and upper bounds ICER (US$115-US$266 over five years and US$111-US$231 over 10 years).

From Fig 2, we can see that when considering provider and user costs, the ICER is slightly lower, which is explained by the fact that averted costs include a higher proportion of user costs.

**Fig 3:**
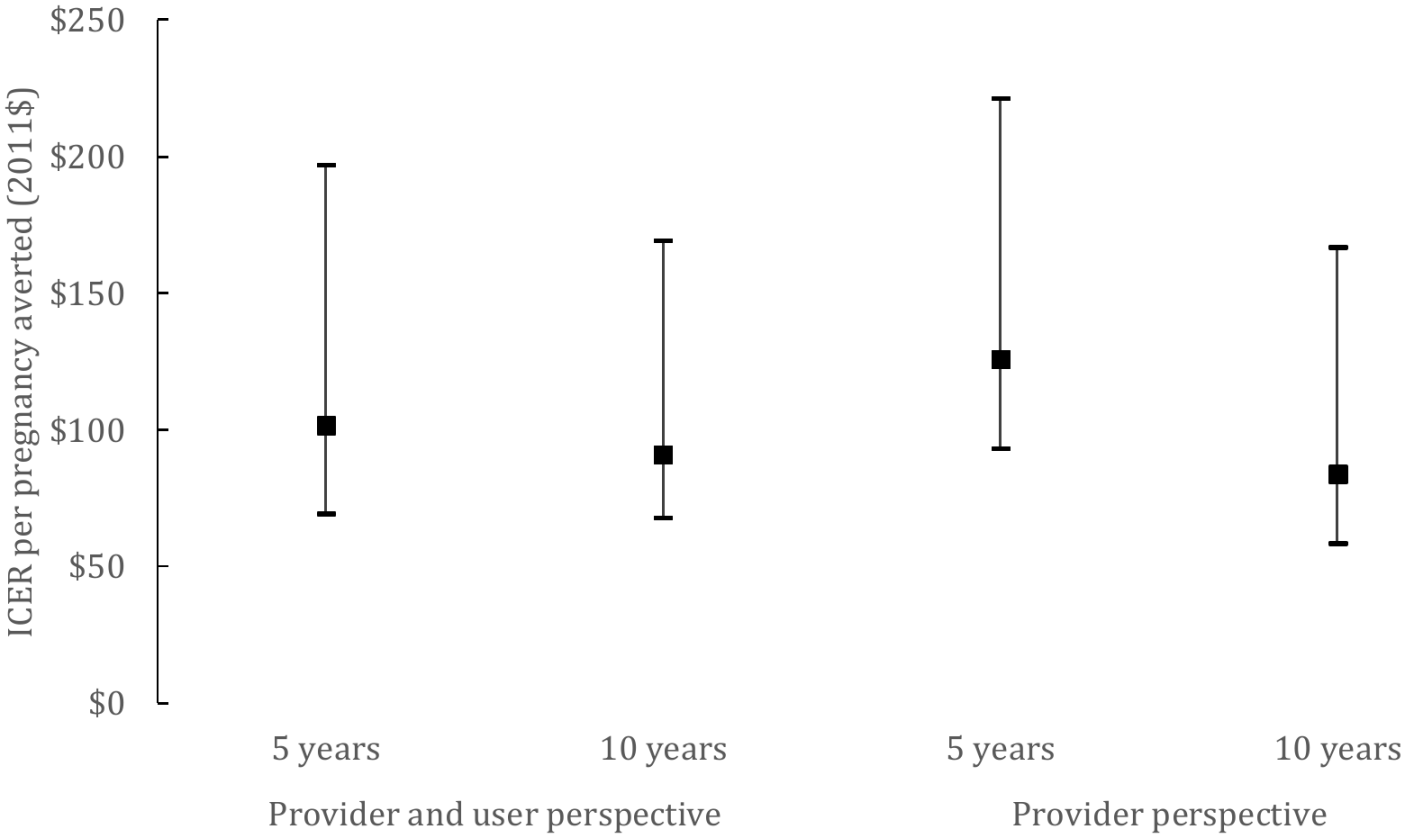
Fig 3 presents the ICER per pregnancy averted in 2011 USD assuming a perfect use.

Finally, when we consider perfect use (Fig 3), the ICER is lower given that the higher adherence leads to improved outcomes in terms of unintended pregnancy, with no extra cost. At the provider level, the introduction of the SILCS diaphragm would cost US$126 per pregnancy averted (US$93-US$221) at five years and US$84 per pregnancy averted (US$58-US$167) at 10 years, while considering user costs, the costs of introducing SILCS diaphragm would be US$102 (US$69-US$197) and US$91 (US$68-US$169) per pregnancy averted over five years and over 10 years, respectively.

**Fig 4:**
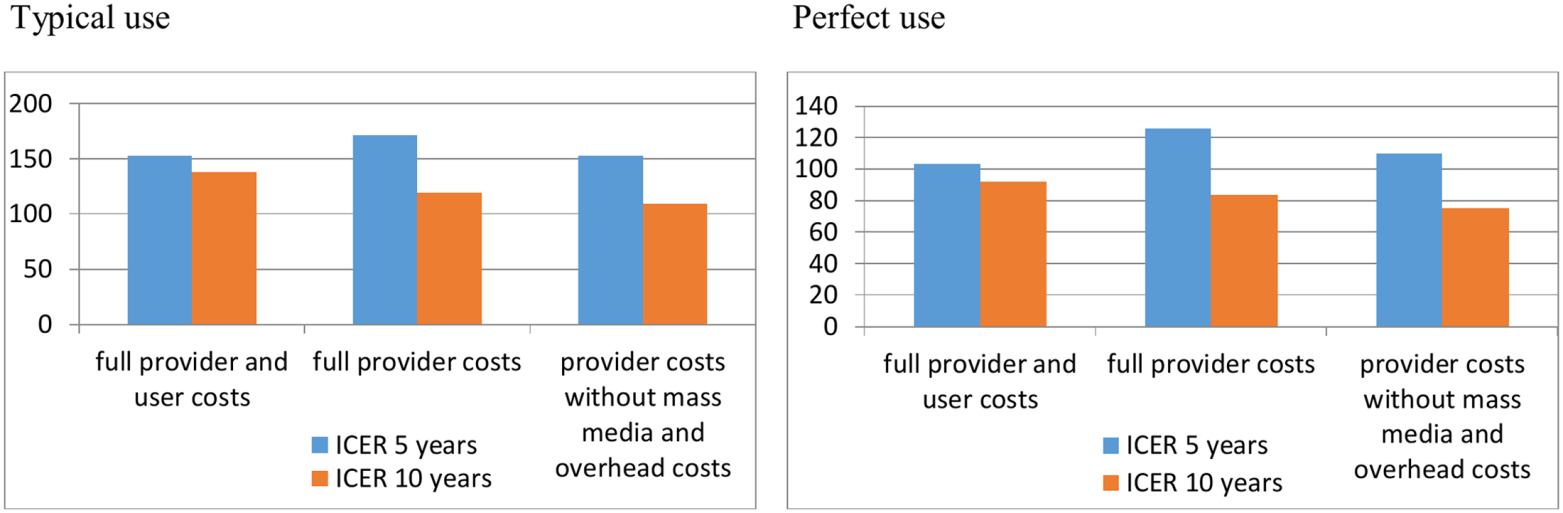
Fig 4 presents the ICER per pregnancy averted in US$2011 depending on the costs considered in the model.

We conducted a univariate sensitivity analysis to determine which variables have the strongest impact on the ICER. The cost of the contraceptive gel has the greatest effect on the cost-effectiveness of SILCS. We can see from Fig 4 that if we do not consider the cost of the mass media campaign and overhead costs (i.e. health systems and facility markups), the ICER only slightly decreases. To achieve cost saving considering user costs under the typical use scenario, the gel price would need to be reduced by 71% (or US$2.62 per tube).

**Fig 5:**
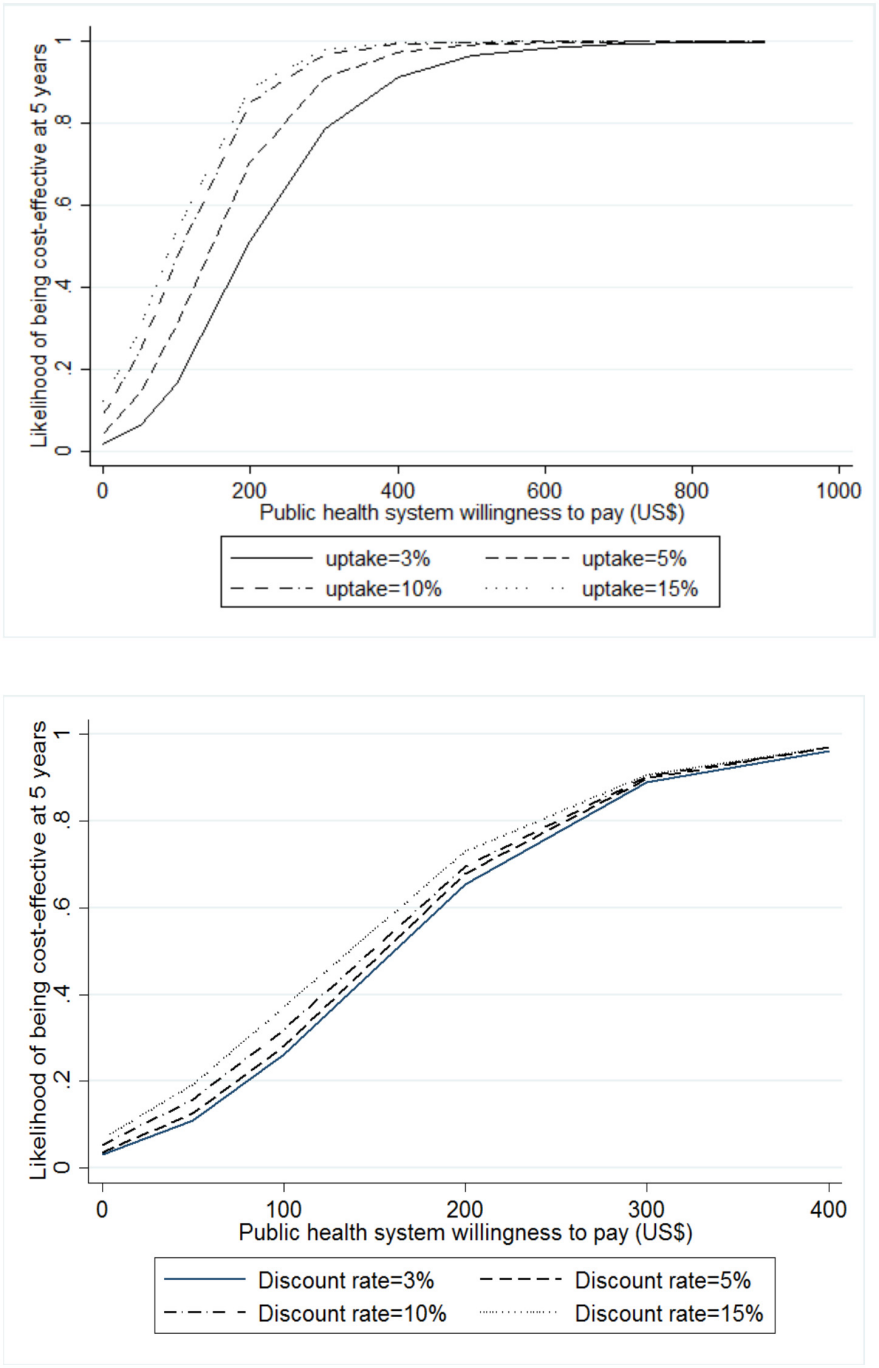
Fig 5 depicts the likelihood that the introduction of SILCS is cost-effective depending on the public health system willingness to pay for different levels of uptake and discount rates.

Cost-effectiveness acceptability curves were constructed to evaluate the probability that the intervention is cost-effective for a range of willingness to pay (WTP) threshold values. We conducted a PSA using 10,000 iterations to investigate the uncertainty in the main model parameters (see S2 Table) on the likelihood of the intervention being cost-effective when considering user costs. When the standard error was not available, we assumed it was equal to 10% of the mean. Alternative scenarios explored the influence of plausible values of uptake and discount rates (varying both from 3% to 15%) on the probability of cost-effectiveness. Fig 5 shows that variations in the discount rate applied to costs and effects leads to little variation in the estimated cost-effectiveness. In contrast, uptake has a very strong negative effect on the ICER, a higher level of uptake allowing the fixed costs of the intervention to be distributed over a greater number of unwanted pregnancies averted. However, since no WTP threshold for pregnancy averted was identified in the literature for South Africa, the likelihood of the intervention of being cost-effective is unknown.

## Discussion

Under a typical use assumption, the introduction of the SILCS diaphragm in Gauteng comes to a cost per pregnancy averted of US$153 and US$171 at five years from a user’s and provider’s perspectives, respectively. Under a typical use assumption, the introduction of the SILCS diaphragm in Gauteng could prevent an estimated 10,482 and 19,960 unintended pregnancies including 2,117 to 4,032 abortions at five years and 10 years, respectively, if 5% of women with an unmet need for modern contraception used the SILCS diaphragm.

We find that our results are mainly driven by the level of the contraceptive gel cost but are also sensitive to the presence of the mass media campaign and training costs. Further work should be conducted to consider alternative approaches to demand creation, such as interpersonal communication approaches or peer educators versus mass media in stimulating demand and adherence, and to compare the costs of those different options. Over time, training on providing SILCS will no longer need to be a vertical catch-up approach but will be integrated into national provider curriculum. In addition, the gel manufacturer should consider ways to reduce the gel costs. For example, one option could be to transfer contraceptive gel production to a local manufacturer in South Africa. Global price negotiations could also address high prices.

We found little difference between the ICER at five and 10 years: the ICER at 10 years is slightly lower than at five years. The main reasons for this are that (1) benefits in terms of pregnancy averted are immediate and remain constant over time and (2) the share of mass media and training cost is small and represents 14% at five years and 6% at 10 years of total discounted incremental costs. This also provides some explanation of the low effect of the discount rate on the ICER. We report this ICER only to 10 years because we believe that multiple factors would likely have evolved by that time which would render estimates over a longer time horizon increasingly unreliable.

The main limitations of the model come from the fact that the majority of the costs are from the literature with not all costs being equally up to date and/or were obtained from different populations and/or different interventions than the SILCS diaphragm introduction among women of the general Gauteng population.

We also did not include the costs of diaphragm side effects as we do not have information on their treatment costs in South Africa. In the literature, side effects such as urinary tract infection and vaginitis have been reported as associated with use of the traditional diaphragm [27], although it is not clear if these are associated with the diaphragm or with the nonoxynol-9-based spermicide that was used widely in past decades, or their combine use. Data from the SILCS diaphragm clinical studies indicate fewer reports of these effects with the SILCS diaphragm than with other diaphragms [7]; the SILCS diaphragm is designed to reduce risk of pressure on the urethra. We also do not consider women who use the SILCS diaphragm without a contraceptive gel. This could lower contraceptive effectiveness but also would lower the recurring cost significantly. We do not take into account any costs averted for reduced risk of cervical sexually transmitted infections [28] and also do not consider the potential benefit of the SILCS diaphragm used with a microbicide gel for dual protection from unintended pregnancies and HIV—a strategy being evaluated in early-stage clinical studies but beyond the scope of this model. The effectiveness and cost-effectiveness of SILCS on the reduction of HIV/STI transmission will be the subject of a future research. We also did not consider that depending on who will bear the cost (the public-sector provider or the user), user behaviors might change, with consequences for the ICER. For instance, wastage could decrease if users were to pay for contraceptive gel.

Additionally, we have not considered the full cost of unintended pregnancies. In low-income countries, pregnancy is one of the main causes of school dropout among schoolgirls [29] and affects women’s future income as well as the nation’s human capital. Net cost of children is defined as the present value of expected outlays plus the imputed value of the parents’ services, minus the present value of the expected money return, plus the imputed value of the child’s services [30]. There is evidence that unplanned pregnancies have a short-term effect on poverty, labor participation, and welfare for some mothers [31]. Due to the lack of data regarding cost and monetary returns of unplanned children in South Africa and given that there is some evidence that those short-term effects dissipate over time [31], we do not consider this element. Psychological and economic costs for the mother resulting from an unintended pregnancy were also not considered.

Finally, a central assumption of the model is that the intervention only reaches women with unmet need for contraception. In reality, some women could switch to SILCS from more effective methods, which could result in an increase in unintended pregnancies. Our results thus provide an optimistic scenario since the effects are maximized under our assumption. However, this assumption reflects the fact that SILCS diaphragm should be seen as a way to reach women with an unmet need for contraception rather than a competitor to existing modern contraceptives. In fact, building on previous literature on the barriers to modern contraception, our model assumed that the high proportion of women with unmet need for contraception was due to the low preference for available contraceptives: the use of hormonal methods being limited by side effects [1] while the use of condom was limited by its association with HIV/AIDS and low women’s bargaining power [4]. Findings from focus group discussions and interviews in KwaZulu Natal conducted as part of a separate health systems assessment exploring opportunities for the introduction of the SILCS diaphragm suggest there is interest in the SILCS diaphragm among women and family planning providers in South Africa and the policy and service delivery environment is supportive [32]. Market research conducted among women of diverse age, racial group, and socioeconomic status from three cities in South Africa suggests a potential—but as of yet undeveloped—market exists, with 5–10% of respondents saying they would definitely purchase SILCS. Future empirical research is needed to assess likely levels of substitution (for example using discrete choice experiments), as in reality, once introduced, it will not be possible to fully avoid substitution. In addition, a higher percent of women expressed interest in SILCS when the concept was introduced as a multipurpose prevention technology that could protect from both unintended pregnancy and HIV (PATH, unpublished report, December 2013). Additional studies are planned in South Africa that will assess preference for SILCS used with a microbicide gel for multipurpose prevention compared to other HIV prevention strategies. This discrete choice experiment will feed into modeling to estimate the potential uptake, impact, and cost-effectiveness of SILCS. Our results and the findings from these other activities will be used to refine the value proposition and introduction strategies for the SILCS diaphragm.

Gauteng region was chosen due to the availability of all the model parameters. Since we have assumed a linear trend for population projection in the Gauteng region and given that total unit cost and cost-effectiveness of the SILCS diaphragm is unlikely to vary greatly between South African regions, our results are assumed to have a relative good external validity at the country level, within the specific population of women in a union. We find that a pregnancy averted using the SILCS diaphragm in the Gauteng region would cost more than those reported by previous studies that assumed use of a diaphragm. In the United States, Chiou et al. find that the ICERs of contraceptives vary between US$16.65 and US$45.35 per pregnancy averted depending on the contraceptive, and it would cost US$32.88 per pregnancy averted for the diaphragm [33]. Using a similar model with typical use, Trussell et al. find a cost of US$25.31 per pregnancy averted by diaphragm use [10]. A main explanation for this difference of results is that the costs required to implement the intervention were not considered in these studies. For comparability, we remove cost categories not previously included (overhead costs, mass media, and training costs), and show our estimates to be US$85 at the provider level considering typical use over five years and US$33 when considering user costs. However, policymakers need information on the full cost of introducing a new contraceptive technology and, therefore, we believe that the full cost should be presented.

We can compare the SILCS diaphragm intervention with other types of interventions to reduce the number of unintended pregnancies. In Kenya, Duflo et al. find that a “sugar daddy talk” that provides information on the risks of having sex with older men would cost US$91 per pregnancy averted [34], training a teacher would cost US$525 per pregnancy averted, and distributing free school uniforms would cost US$750 per pregnancy averted. Then it seems that the SILCS diaphragm would have a lower ICER than other types of interventions (e.g. behavioral interventions) that reduce unintended pregnancies.

## Conclusion

Reducing the number of women with unmet need for contraception is a challenge for most African governments. There is a great need for access to contraceptive technologies that can meet the needs of women who are not using existing methods, in part because they want a user-initiated method or have concerns about side effects. We analyzed the cost-effectiveness of the introduction of the SILCS diaphragm in the Gauteng region in South Africa among women with unmet need compared to no contraception for this population. We found that at five years using the provider’s perspective the introduction of the SILCS diaphragm would cost between US$125 and US$171 per pregnancy averted depending on adherence, while when considering user costs, the ICER per averted pregnancy would be US$103 to US$153. These results are especially sensitive to the proportion of women with unmet need targeted by the intervention. Additionally, costs are strongly sensitive to the cost of the contraceptive gel and slightly sensitive to the mass media and direct programming costs of the SILCS diaphragm. Depending on the government’s willingness to pay and its other options to prevent pregnancies, the SILCS diaphragm could be an attractive contraceptive method to reduce unmet need for contraception.

## Supporting Information

S1 Fig Composition of costs (excluding averted costs). (DOCX)

S1 Table Value of parameters per year. (DOCX)

S2 Table Parameters with uncertainty estimated per year considering a time horizon of 5 years and user costs. (DOCX)

S3 Table Details of costs and impacts at 5 and 10 years (US$ 2011). (DOCX)

S1 Text Literature review. (DOCX)

S2 Text Targeted population projections. (DOCX)
