# Supplementary material for: Cost-Effectiveness of Introducing the SILCS Diaphragm in South Africa
Source: PLoS One. 2015 Aug 21;10(8):e0134510. doi: 10.1371/journal.pone.0134510 (PMC4546642; doi:10.1371/journal.pone.0134510)
Supplement: S1 Table — (DOCX) [file pone.0134510.s002.docx]

S1 Table: Value of parameters per year

| **Parameter** | **Value** | **Source** |
| --- | --- | --- |
| **Environmental parameters** |  |  |
| Discount rate in South Africa | 3% | [33] |
| % living in rural area | 3% | [1] |
| % living in urban area | 97% | [1] |
| Number of public health facilities in Gauteng | 295 | [10] |
| Product life cycle (in years) | 2 | [6] |
| Number of sex acts per year | 104 | [1] |
| Quantity of gel used per sex (in ml) | 4 | Kessel, personal communication |
| Wastage contraceptive gel | 10% | [12] |
| Duration of counseling for using SILCS per visit (in hours) | 0.5 | [34] |
| Share of refresher training cost in total training cost | 30% | [13] |
| Frequency of refresher training cost (in years) | 3 | [13] |
| Average number of days of work lost due to pregnancy | 3 | Assumed |
| Duration of prenatal care visit (hours) | 0.5 |  |
| Number of prenatal visits per pregnancy | 4 | [1] |
| Proportion of births that are really unwanted | 49% | [1] |
| Proportions of unwanted births that are mistimed | 51% | [1] |
| Years when mistimed births would occur | 2 | [8] |
| Abortion rate in Gauteng | 10.5% | [24] |
| Abortion rate among unintended pregnancies | 20.20% | Computed from DHS 2003 |
| Delivery rate among unintended pregnancies | 79.80% | Computed from DHS 2003 |
| Number of visits for abortion (pre-act) | 3 | [35] |
| % of women receiving antenatal care | 92% | [1] |
| % of women where delivery was assisted by skilled health worker | 92% | [1] |
| Duration of prenatal care visit (in hours) | 0.5 | [36] |
| Number of prenatal visits needed when pregnant | 4 | [1] |
| **Unit cost (2011US$)** |  |  |
| Unit cost SILCS per user | 54.5 | [7] |
| Unit cost delivery and antenatal care (facility) | 265.7 | Computed from the model |
| Unit cost abortion (facility) | 125.9 | Computed from the model |
| **Effect** |  |  |
| Likelihood of getting pregnant without any contraception | 40% | [1] |
| Efficacy of diaphragm: |  |  |
| Typical usage (%) | 17.8% | [7] |
| Perfect usage (%) | 13.7% | [7] |
