# Supplementary material for: Cost-Effectiveness of Introducing the SILCS Diaphragm in South Africa
Source: PLoS One. 2015 Aug 21;10(8):e0134510. doi: 10.1371/journal.pone.0134510 (PMC4546642; doi:10.1371/journal.pone.0134510)
Supplement: S2 Table — (DOCX) [file pone.0134510.s003.docx]

S2 Table: Parameters with uncertainty estimated per year considering a time horizon of 5 years and user costs^e^

| **Proportions and probabilities:** | **mean** | **standard error** | **distribution** | **alpha** | **beta** |
| --- | --- | --- | --- | --- | --- |
| Likelihood of getting pregnant without contraception | 0.4 | 0.04 | beta | 59.600 | 89.4 |
| Likelihood of getting pregnant with SILCS | 0.178 | 0.018 | beta | 82.022 | 378.776 |
| Proportion of women aged 15-49 sexually active | 0.46 | 0.006 | beta | 3182.080 | 3769.91 |
| Proportion of South Africans living in Gauteng | 0.23 | 0.047 | beta | 18.952 | 62.528 |
| Proportion of women with unmet need for modern contraception | 0.13 | 0.05 | beta | 5.834 | 38.701 |
| Proportion of mistimed births | 0.51 | 0.159 | beta | 4.537 | 4.393 |
| Proportion of unwanted births | 0.49 | 0.159 | beta | 4.393 | 4.537 |
| Proportion of abortions in Gauteng | 0.105 | 0.011 | beta | 89.395 | 761.986 |
| **Cost:** |  |  |  |  |  |
| Cost delivery and antenatal care | 238.738 | 23.874 | gamma | 100 | 2.387 |
| Cost of an unwanted pregnancy | 188.73 | 18.873 | gamma | 100 | 1.887 |
| Cost of an abortion | 175.62 | 17.561 | gamma | 100 | 1.756 |
| SILCS yearly overhead and training costs | 181435 | 18143.5 | gamma | 100 | 1814.35 |
| Yearly product cost | 54.559 | 5.456 | gamma | 100 | 0.546 |
| **Environmental parameters:** |  |  |  |  |  |
| Year when mistimed birth will occur | 2 | 0.2 | gamma | 100 | 0.02 |
| Projection of population of women in South Africa | 14138.7 | 1413.872 | gamma | 100 | 141.387 |
