# Supplementary material for: Cost-Effectiveness of Introducing the SILCS Diaphragm in South Africa
Source: PLoS One. 2015 Aug 21;10(8):e0134510. doi: 10.1371/journal.pone.0134510 (PMC4546642; doi:10.1371/journal.pone.0134510)
Supplement: S3 Table — (DOCX) [file pone.0134510.s004.docx]

S3 Table: Details of costs and impacts at 5 and 10 years (US$ 2011)

|  | 5 years | 10 years |
| --- | --- | --- |
| **IMPACTS** |  |  |
| **Projections of users in Gauteng** |  |  |
| Medium fertility/median (median) | 50,164 | 102,991 |
| Low fertility/lower bound 95% uncertainty (lower bound) | 21,574 | 38,559 |
| Constant fertility/upper bound 95% uncertainty (upper bound) | 92,322 | 202,300 |
| **Pregnancies averted using typical usage** |  |  |
| Medium fertility/median (median) | 10,482 | 19,960 |
| Low fertility/lower bound 95% uncertainty (lower bound) | 4,519 | 7,580 |
| Constant fertility/upper bound 95% uncertainty (upper bound) | 19,262 | 38,964 |
| **COSTS** |  |  |
| **Provider costs excluding averted costs** |  |  |
| Medium fertility/median (median) | 3,037,410 | 4,595,066 |
| Low fertility/lower bound 95% uncertainty (lower bound) | 1,821,352 | 2,506,599 |
| Constant fertility/upper bound 95% uncertainty (upper bound) | 4,826,026 | 7,773,775 |
| **Provider costs including averted costs** |  |  |
| Medium fertility/median (median) | 1,792,695 | 2,383,912 |
| Low fertility/lower bound 95% uncertainty (lower bound) | 1,283,377 | 1,655,179 |
| Constant fertility/upper bound 95% uncertainty (upper bound) | 2,542,092 | 3,483,893 |
| **Provider costs excluding averted costs per user** |  |  |
| Medium fertility/median (median) | 61 | 45 |
| Low fertility/lower bound 95% uncertainty (lower bound) | 84 | 65 |
| Constant fertility/upper bound 95% uncertainty (upper bound) | 52 | 38 |
| **Provider costs including averted costs per user** |  |  |
| Medium fertility/median (median) | 36 | 23 |
| Low fertility/lower bound 95% uncertainty (lower bound) | 59 | 43 |
| Constant fertility/upper bound 95% uncertainty (upper bound) | 28 | 17 |
| **ICER with provider perspective including averted costs** |  |  |
| Medium fertility/median (median) | 171 | 119 |
| Low fertility/lower bound 95% uncertainty (lower bound) | 284 | 218 |
| Constant fertility/upper bound 95% uncertainty (upper bound) | 132 | 89 |
| **Provider and user costs excluding averted costs** |  |  |
| Medium fertility/median (median) | 3,473,475 | 6,072,257 |
| Low fertility/lower bound 95% uncertainty (lower bound) | 2,008,093 | 3,029,777 |
| Constant fertility/upper bound 95% uncertainty (upper bound) | 5,631,268 | 10,742,736 |
| **Provider and user costs including averted costs** |  |  |
| Medium fertility/median (median) | 1,608,096 | 2,758,536 |
| Low fertility/lower bound 95% uncertainty (lower bound) | 1,201,862 | 1,753,806 |
| Constant fertility/upper bound 95% uncertainty (upper bound) | 2,208,475 | 4,313,752 |
| **Provider and user costs excluding averted costs per user** |  |  |
| Medium fertility/median (median) | 69 | 59 |
| Low fertility/lower bound 95% uncertainty (lower bound) | 93 | 79 |
| Constant fertility/upper bound 95% uncertainty (upper bound) | 61 | 53 |
| **Provider and user costs including averted costs per user** |  |  |
| Medium fertility/median (median) | 32 | 27 |
| Low fertility/lower bound 95% uncertainty (lower bound) | 56 | 45 |
| Constant fertility/upper bound 95% uncertainty (upper bound) | 24 | 21 |
| **ICER with Provider and user perspective including averted costs** |  |  |
| Medium fertility/median (median) | 153 | 138 |
| Low fertility/lower bound 95% uncertainty (lower bound) | 266 | 231 |
| Constant fertility/upper bound 95% uncertainty (upper bound) | 115 | 111 |
