# Supplementary material for: Cost-Effectiveness of Introducing the SILCS Diaphragm in South Africa
Source: PLoS One. 2015 Aug 21;10(8):e0134510. doi: 10.1371/journal.pone.0134510 (PMC4546642; doi:10.1371/journal.pone.0134510)
Supplement: S1 Text — (DOCX) [file pone.0134510.s005.docx]

S1 Text: Literature review

Costs were estimated from the literature. The databases searched were Pubmed, Embase, and Google Scholar. The following terms were used to construct the search. :**“HIV”, “AIDS”, “sexually transmitted disease”, “sexually transmitted infection”, “STD”, “STI”, “cost”, “cost benefit”, “economic”, “cost effective*”, “evaluation”, “acceptability”, “diaphragm”, “SILCS”, “contracept*”, “Microbicide delivery”, “Cervical”, “Family planning services”, ”Reproductive Health Intervention”, ”PMTCT”, “prevention of mother to child transmission”**, **“Preventi*”**, **“ART”**, “**antiretroviral therapy”, “testing”, “Sex workers”**, **“female”**, **“woman”**, **“pregnan*****”**. Bibliographies of relevant studies were hand searched and examined through a “snowball sampling” process in order to identify additional costing-related studies and references. Database searches were restricted to studies written in English, located in South Africa, and published between January 2006 and June 2013. Papers published prior to 2010 were used to identify more recent citations. Papers were checked for eligibility by reading through the title and abstract. Studies were included if there were costing or quantitative descriptive data available in relation to provider costs (facility delivery costs, training costs, mass media costs), user costs (transport costs, opportunity cost of time), and averted costs (averted cost at the provider level). Studies were also included if the outcome was pregnancy or HIV prevention averted. The search identified 33 potentially relevant articles that were in English and published in or after 2006 in South Africa. Out of these studies, 9 studies had costing information relevant to our search. SILCS product and contraceptive gel unit costs were obtained from PATH.
