# Supplementary material for: Cost-Effectiveness of Introducing the SILCS Diaphragm in South Africa
Source: PLoS One. 2015 Aug 21;10(8):e0134510. doi: 10.1371/journal.pone.0134510 (PMC4546642; doi:10.1371/journal.pone.0134510)
Supplement: S2 Text — (DOCX) [file pone.0134510.s006.docx]

S2 Text: Targeted population projections

First, projections of the number of women aged between 15-49 years until 2030 was obtained from the United Nations [14]. Different models are available depending on assumption regarding fertility (medium, high, low, and constant), instant replacement, zero migration, constant mortality, and no change. We present the results with medium fertility and with the lowest (low fertility) and highest (constant fertility) bound in order to see how sensitive the results are to a change in assumption on population growth trend. We focus on sexually active women (45.77% of women of 15-49 years according to DHS 2003). We then applied the prediction of the United Nations regarding the proportion of women with unmet need for contraception in South Africa. The predictions are conducted using the 1998 and 2003 DHS and are predicted until 2015 for three different values, median value as well as the upper and lower bounds of the 95% confidence intervals. Linear functional forms were used to extend the model until 2030. Predictions contain nine scenarios that combine two elements, fertility scenario to predict population growth (medium, low, and constant) and scenario used to predict unmet need for modern contraception (median, upper, and lower bound). We present the results using (1) medium fertility and median unmet need assumptions, (2) low fertility and upper bound 95% uncertainty as the upper bound, and (3) constant fertility and lower bound 95% uncertainty for the lower bound. Finally, in order to predict the uptake of diaphragms among the women with unmet need for contraception, we assumed that 5% of women with unmet need for contraception will choose the diaphragm among other possible contraceptive methods. We further conducted a PSA on this parameter.
